# “Preventing vision loss in pediatric otogenic thrombosis: a case report highlighting surgical interventions”

**DOI:** 10.3389/fopht.2026.1736235

**Published:** 2026-02-25

**Authors:** Fatimah A. AlMuhanna, Wejdan S. Hakami, Abdullah M. AlHashem, Eman AlShahwan, Yazeed A. AlHarbi, Shatha S. AlShafi

**Affiliations:** 1 Division of Pediatric Neurology, Department of Pediatrics, Prince Sultan Military Medical City, Riyadh, Saudi Arabia; 2 Division of Neuroradiology, Department of Radiology, Prince Sultan Military Medical City, Riyadh, Saudi Arabia; 3 Division of Neuro-Ophthalmology, Department of Ophthalmology, Prince Sultan Military Medical City, Riyadh, Saudi Arabia; 4 Department of Ophthalmology, Magrabi Health, Riyadh, Saudi Arabia

**Keywords:** intracranial hypertension, lumbar drainage, optic nerve sheath fenestration, otogenic CVST, pediatric papilledema

## Abstract

Otogenic cerebral venous sinus thrombosis (CVST) is a rare complication of acute otitis media in children and may lead to severe intracranial hypertension with vision-threatening papilledema. We report the case of a 4-year-old girl with otogenic CVST involving the left transverse and sigmoid sinuses, complicated by marked intracranial hypertension and bilateral Frisén grade 4 papilledema. Serial ophthalmologic assessments and neuroimaging were performed to evaluate the response to sequential therapeutic interventions. Despite treatment with intravenous antibiotics, anticoagulation, and medical intracranial pressure–lowering therapy, papilledema and symptoms persisted. Optic nerve sheath fenestration (ONSF) resulted in partial improvement; however, subsequent temporary external lumbar drainage led to rapid and sustained resolution of papilledema, headache, and left abducens nerve palsy, with preservation of visual function. This case underscores the importance of a structured, stepwise escalation strategy incorporating both vision-directed and global intracranial pressure–lowering interventions when medical therapy alone is insufficient.

## Introduction

Acute otitis media is among the most common infectious conditions in childhood, with approximately 60% of children experiencing at least one episode by age three (1). Although uncommon, it may progress to otogenic cerebral venous sinus thrombosis (CVST), a serious intracranial complication with a reported prevalence between 0.2% and 2.7% (2). Thrombosis of the transverse or sigmoid sinus impairs venous outflow and cerebrospinal fluid (CSF) absorption, leading to elevated intracranial pressure (ICP), papilledema, and risk of permanent visual loss (3, 4).

While medical therapy with antibiotics, anticoagulation, and ICP-lowering agents remains first-line treatment, guidance regarding surgical escalation for vision preservation is limited (5, 6).Optic nerve sheath fenestration (ONSF) and CSF diversion procedures have been reported individually (7, 8), but their sequential use in pediatric otogenic CVST is not well defined (9, 10).

We report a pediatric case of otogenic cerebral venous sinus thrombosis with severe intracranial hypertension and vision-threatening papilledema, illustrating a stepwise neuro-ophthalmic management approach using optic nerve sheath fenestration followed by temporary lumbar drainage. This case emphasizes the role of timely, multidisciplinary intervention in preserving visual function when medical therapy alone is inadequate.

## Case description

### Clinical presentation

A previously healthy 4-year-old girl presented with a 3-day history of fever and left otalgia, followed by progressive headache, vomiting, photophobia, diplopia, and neck discomfort.

### Clinical findings

Neuro-ophthalmologic examination demonstrated severe bilateral papilledema (Frisén grade 4 OU) accompanied by a left abducens nerve palsy. Assessment of visual acuity was limited by patient discomfort and was confined to qualitative measures, revealing finger counting at close distance in the right eye and hand-motion perception in the left eye; reliable quantitative acuity testing was not feasible. Fixation and tracking were preserved, and pupillary responses were symmetric without a relative afferent pupillary defect. Formal visual field testing was not performed due to the patient’s age and discomfort, while optical coherence tomography could not be obtained at presentation because of limited patient cooperation.

## Diagnostic assessment

**Figure 1:**
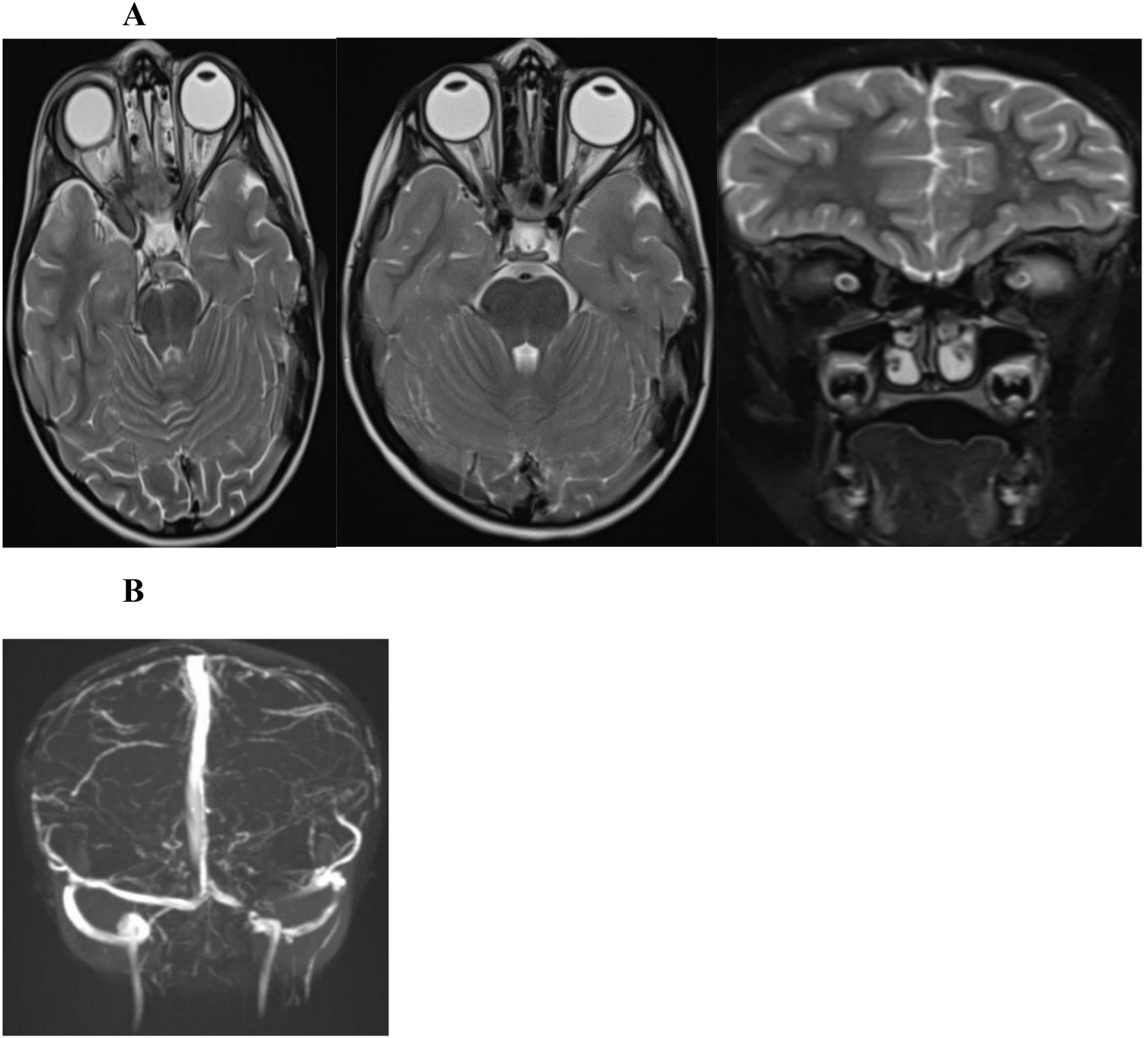
Baseline neuroimaging prior to treatment. **(A)** 2 Axial and one coronal T2-weighted MRI of the brain and orbits at the level of optic nerve heads, demonstrate dilatation of the optic nerve sheath complexes with tenting of the optic nerve heads and flattening of the posterior globes, consistent with elevated intracranial pressure. **(B)** MR venography demonstrates thrombosis of the left transverse sinus, sigmoid sinus, and jugular bulb, with non-enhancing filling defects.

Lumbar puncture demonstrated an opening pressure of 42 cm H_2_O with normal CSF cell counts and sterile cultures. Contrast-enhanced CT venography and MR venography confirmed complete thrombosis of the left transverse and sigmoid sinuses extending into the jugular bulb. MRI showed optic nerve sheath distension and posterior scleral flattening, consistent with elevated ICP (**Figure 1**).

## Therapeutic interventions

### Medical management

The patient was managed with intravenous ceftriaxone and vancomycin, subcutaneous low-molecular-weight heparin (20 mg twice daily; anti-Xa target 0.75–1.0), and acetazolamide, titrated to a maximum dose of 25 mg/kg/day. Furosemide (1 mg/kg/day) was subsequently added. Although the left abducens nerve palsy improved, headache persisted, and papilledema remained severe on follow-up examination (Frisén grade 3 in the right eye and grade 4 in the left eye).

### Optic nerve sheath fenestration

Optic nerve sheath fenestration was performed on hospital day 21 due to persistent, vision-threatening papilledema refractory to maximal medical therapy. On preoperative examination (day 20), papilledema was graded as grade 3 in the right eye and grade 4 in the left eye. By postoperative day 2 (day 23), papilledema in the left eye had improved to grade 3.

### Lumbar drain placement

Given the lack of adequate clinical and ophthalmic improvement in papilledema, headache, and left sixth cranial nerve palsy despite maximal medical therapy and prior optic nerve sheath fenestration consistent with persistent intracranial hypertension, a temporary lumbar drain was placed for a total duration of six days. The opening cerebrospinal fluid (CSF) pressure was elevated at 38 cm H_2_O. Continuous CSF drainage was performed at a rate of 5–10 mL/hour under strict aseptic conditions. No procedure-related complications were encountered.

Following CSF diversion, there was a marked clinical and ophthalmic response. Papilledema improved to Frisén grade 1 bilaterally, with complete resolution of headache and left sixth cranial nerve palsy.

### Follow-up and outcomes

At discharge (week 6), the patient was clinically stable and discharged on ongoing anticoagulation therapy, with acetazolamide and furosemide gradually tapered. Ophthalmologic examination at that time demonstrated residual mild papilledema (Frisén grade 1 OU), with complete resolution of headache and left abducens nerve palsy.

**Figure 2:**
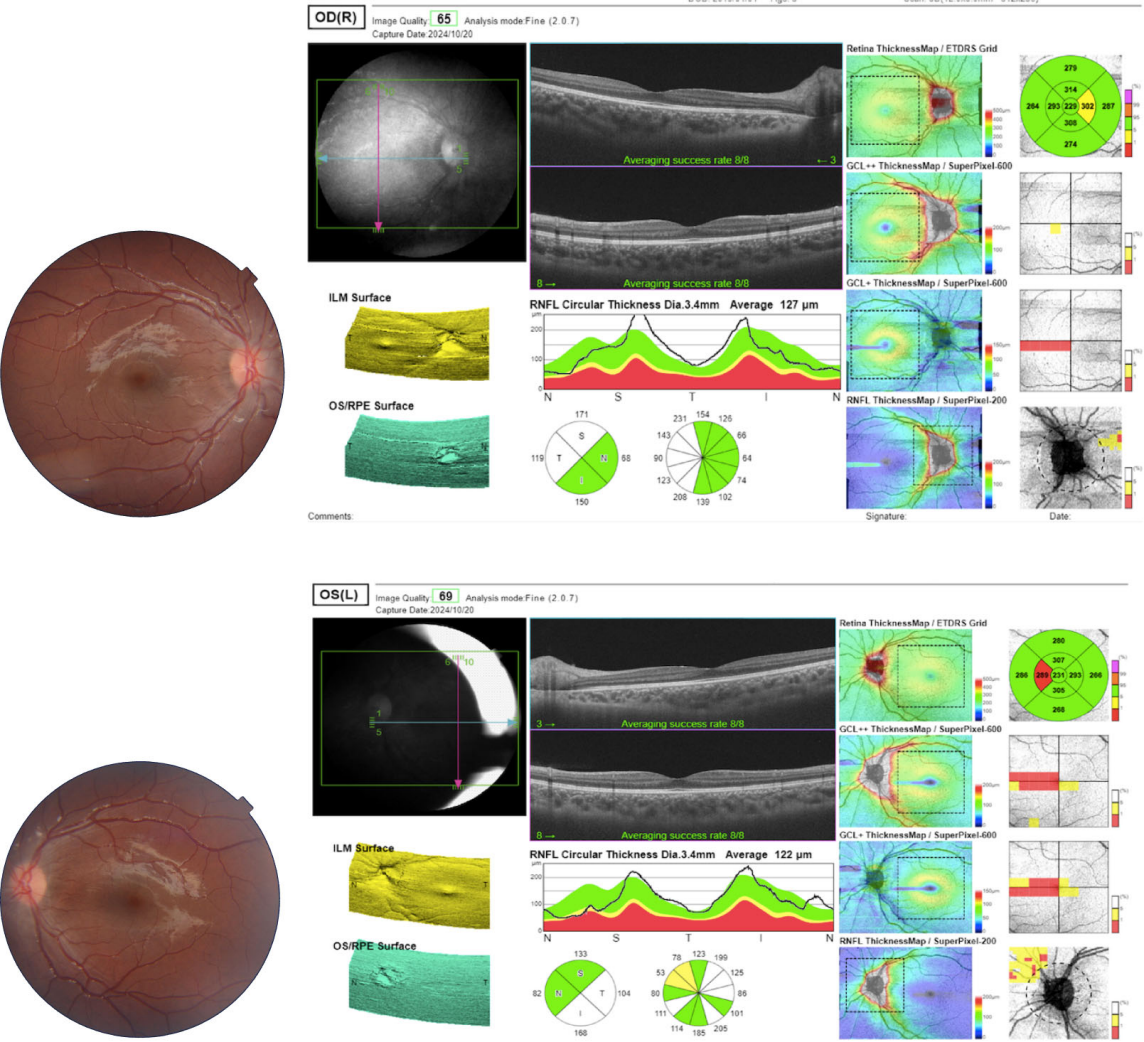
Ophthalmologic findings at two-month follow-up. Fundoscopic examination demonstrates complete resolution of optic disc edema in both eyes following left optic nerve sheath fenestration and temporary lumbar drainage. Optical coherence tomography shows near-normalization of retinal nerve fiber layer thickness, consistent with resolving papilledema.

**Figure 3:**
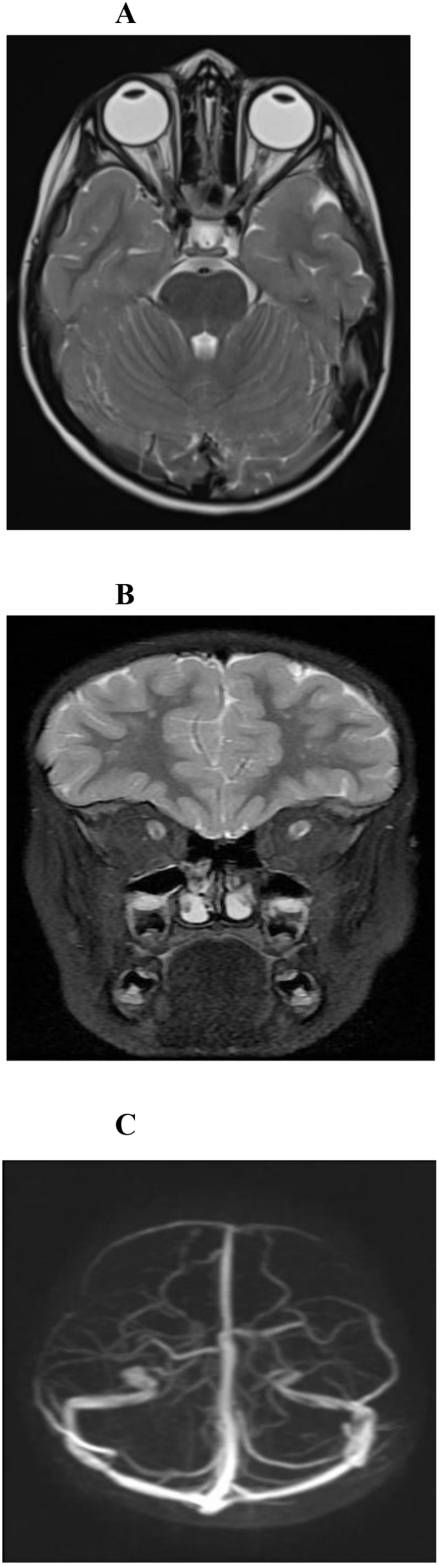
Follow-up neuroimaging after treatment. **(A, B)** Axial and coronal T2-weighted MRI of the brain and orbits at the level of optic nerve heads obtained after optic nerve sheath fenestration and lumbar puncture demonstrate a marked reduction in optic nerve sheath dilatation, with improvement in posterior scleral flattening, consistent with resolution of elevated intracranial pressure. Interval improvement is evident on the most recent imaging **(B)** compared with earlier post-treatment imaging **(A, C)** MR venography demonstrates partial recanalization of the left transverse sinus with improved opacification of the left sigmoid sinus and jugular bulb.

At the two-month follow-up, the patient showed complete clinical and ophthalmologic recovery. Fundoscopic examination confirmed full resolution of papilledema. Optical coherence tomography (OCT) demonstrated normalization of retinal nerve fiber layer (RNFL) thickness bilaterally (OD: 127 µm; OS: 122 µm; **Figure 2**). Brain magnetic resonance imaging (MRI) revealed restoration of posterior globe concavity with reduction in optic nerve sheath diameter (**Figure 3**), while magnetic resonance venography (MRV) showed partial recanalization of the left transverse and sigmoid sinuses. Clinically, the patient reported complete resolution of headache, and neurological examination confirmed full recovery of the left abducens nerve palsy.

**Figure 4:**
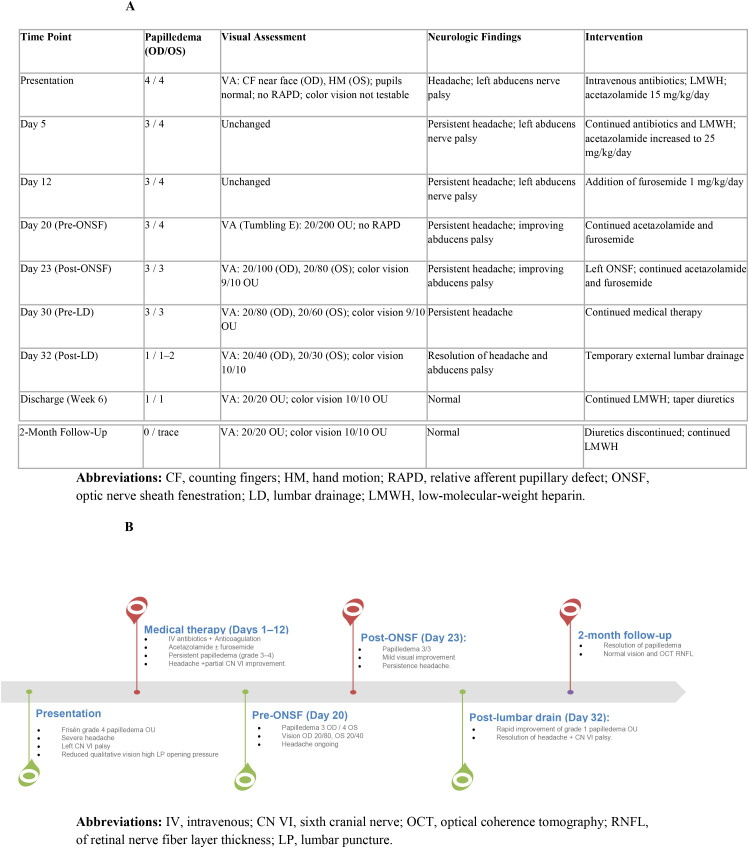
Ophthalmologic course and stepwise therapeutic escalation. **(A)** Serial ophthalmologic examinations and interventions from presentation through two-month follow-up. **(B)** Schematic timeline illustrating stepwise escalation from medical therapy to optic nerve sheath fenestration and subsequent temporary lumbar drainage, resulting in complete resolution of papilledema with full recovery of visual function and headache. CF, counting fingers; HM, hand motion; RAPD, relative afferent pupillary defect; ONSF, optic nerve sheath fenestration; LD, lumbar drainage; LMWH, low-molecular-weight heparin. IV, intravenous; CN VI, sixth cranial nerve; OCT, optical coherence tomography; RNFL, of retinal nerve fiber layer thickness; LP, lumbar puncture.

Low-molecular-weight heparin was continued for a total duration of approximately three months, given the provoked nature of the cerebral venous sinus thrombosis and the absence of clinical or radiological progression. A consolidated overview of the clinical course, therapeutic interventions, and ophthalmologic outcomes is presented in the timeline (**Figure 4**).

## Discussion

Otogenic cerebral venous sinus thrombosis (CVST) is an uncommon but potentially life-threatening complication of acute otitis media in children, with reported mortality reaching 5–10% and significant risk for neurological and visual morbidity when diagnosis or treatment is delayed (4). Thrombosis most often involves the transverse and sigmoid sinuses due to their proximity to the mastoid air cells, where local inflammation can promote endothelial injury, platelet activation, and fibrin deposition, resulting in mural thrombus formation and possible propagation into the jugular bulb and petrosal sinuses (5). Impaired venous outflow disrupts cerebrospinal fluid (CSF) absorption, leading to otitic intracranial hypertension characterized by elevated intracranial pressure (ICP) and papilledema.

Clinical symptoms frequently begin with otologic features such as fever, otalgia, and otorrhea, followed by neurological or ophthalmic manifestations related to raised ICP. In pediatric cohorts, papilledema, abducens nerve palsy, diplopia, facial nerve palsy, headache, nausea, lethargy, and seizures are among the most commonly reported findings (11). These features were evident in our patient, whose bilateral severe papilledema and cranial nerve VI palsy reflected substantial intracranial hypertension.

Initial management universally includes broad-spectrum intravenous antibiotics and anticoagulation to prevent thrombus propagation and support restoration of venous drainage (6). Surgical interventions such as mastoidectomy or abscess drainage are generally reserved for cases with persistent infection or progressive intracranial complications despite adequate medical therapy (9). However, management becomes more challenging when visual function is threatened by refractory intracranial hypertension, and consensus regarding the optimal escalation strategy remains limited (12).

In this patient, papilledema persisted at Frisén grade 3–4 despite maximal medical therapy, placing the optic nerves at imminent risk of irreversible injury. Management was therefore guided by previously reported strategies for vision-threatening papilledema in cerebral venous sinus thrombosis (CVST). Optic nerve sheath fenestration (ONSF) was selected as the initial surgical intervention because progressive papilledema and visual risk were the dominant clinical concerns, consistent with published evidence demonstrating that ONSF can stabilize or improve visual outcomes in CVST when medical therapy alone is insufficient (13, 14).

Although ONSF resulted in improvement of papilledema in the operated eye, persistent edema in the contralateral eye indicated ongoing global intracranial pressure (ICP) elevation, a limitation that has been well described in prior studies (13). This incomplete bilateral response was further supported by neuroimaging findings, including optic nerve sheath distension and posterior globe flattening, which are recognized radiologic markers of sustained intracranial hypertension. These findings suggested that, while ONSF effectively protected the optic nerve locally, it did not adequately address the underlying global ICP burden related to persistent venous outflow obstruction.

Consequently, escalation to temporary lumbar drainage was undertaken to achieve systemic CSF diversion, an approach supported in refractory intracranial hypertension as a temporizing measure when permanent CSF diversion is undesirable, particularly in the setting of recent infection (15, 16). In our patient, lumbar drainage resulted in rapid bilateral improvement in papilledema, preventing the need for permanent CSF shunting. This clinical course supports a complementary, stepwise management strategy, in which ONSF serves to protect optic nerve function while lumbar drainage addresses global ICP elevation, in alignment with previously published experience.

This case contributes to the limited pediatric literature by providing detailed sequential ophthalmologic and radiologic documentation, illustrating how targeted interventions can preserve visual function in otogenic CVST with severe ICP elevation. Strengths of the report include high-quality imaging correlation and detailed examination timelines, while limitations include the inability to obtain pre-treatment OCT or formal visual acuity due to young age and symptom severity.

## Patient perspective

The patient’s family expressed concern about potential permanent vision loss but reported feeling reassured by frequent updates and clear explanations of the treatment plan. They noted significant rapid improvement after lumbar drainage and expressed satisfaction with the final visual outcome.

## Data Availability

The original contributions presented in the study are included in the article/supplementary material. Further inquiries can be directed to the corresponding author.
